# Results of concomitant cryoablation for atrial fibrillation during mitral valve surgery

**DOI:** 10.1093/icvts/ivab322

**Published:** 2021-11-13

**Authors:** Alexander Bogachev-Prokophiev, Ravil Sharifulin, Anastasiia Karadzha, Sergey Zheleznev, Alexander Afanasyev, Mikhail Ovcharov, Alexey Pivkin, Anton Zalesov, Sergey Budagaev, Sergey Ivantsov, Alexander Chernyavsky

**Affiliations:** 1 Department of Heart Valve Surgery, E. Meshalkin National Medical Research Centre, Novosibirsk, Russian Federation; 2 Department of Congenital Heart Disease, E. Meshalkin National Medical Research Centre, Novosibirsk, Russian Federation; 3 Department of Aortic and Coronary Artery Surgery, E. Meshalkin National Medical Research Centre, Novosibirsk, Russian Federation

**Keywords:** Mitral valve disease, Atrial fibrillation, Cryoablation

## Abstract

**OBJECTIVES:**

Concomitant atrial fibrillation ablation during mitral valve (MV) surgery using radio frequency energy sources has been reported previously with excellent outcomes. However, data regarding the effectiveness of concomitant cryoablation remain limited. This study aimed to assess the efficacy of concomitant cryoablation in patients scheduled for MV surgery.

**METHODS:**

Between 2012 and 2020, 242 adult patients who underwent MV surgery and concomitant cryoablation were included. Data on rhythm, medication status and clinical events were assessed at 3, 6 and 12 months, then annually thereafter.

**RESULTS:**

Early mortality was 0.4%. The mean follow-up period duration was 43.9 months. The survival rates at 1, 3 and 5 years were 97.3%, 94.3% and 87.7%, respectively. The rates of freedom from atrial arrhythmia paroxysms at 1, 3 and 5 years were 79.0%, 64.0% and 60.5%, respectively. Atrial arrhythmia recurrence was associated with isolated left atrial lesion set (*P* = 0.038), large right atrial size (*P* = 0.002), lower surgeon experience (*P* = 0.003) and atrial fibrillation paroxysms in the early postoperative period (*P* = 0.002).

**CONCLUSIONS:**

Concomitant cryoablation during MV surgery is a safe and reproducible technique. The procedure provides acceptable freedom from atrial arrhythmias recurrences during long-term follow-up. The biatrial lesion set has advantages over the left atrium pattern in terms of atrial arrhythmias freedom. Surgeon experience significantly influences atrial fibrillation ablation success. Randomized trials are needed to compare radiofrequency and cryoablation.

**Figure:**
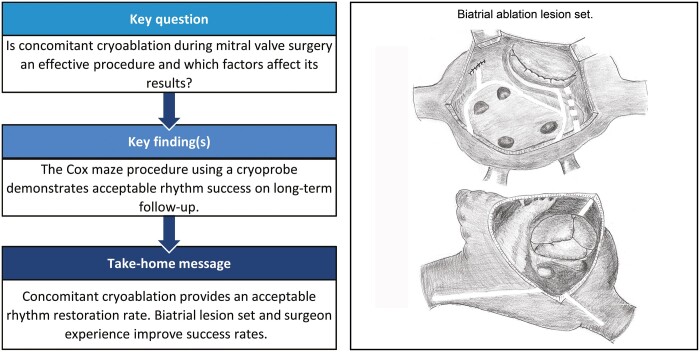

## INTRODUCTION

Atrial fibrillation (AF) is the most common cardiac arrhythmia observed in clinical practice, affecting ∼2% of the European population [1]. AF is associated with an increased risk of cardiovascular mortality, thromboembolic events, sudden cardiac death and heart failure [1]. The ablation procedure is currently the gold standard for the surgical treatment of AF [2, 3]. For a long time, the classical ‘cut and sew’ technique was used, but modern methods using alternative energy sources, such as radiofrequency and cryoablation, are more preferred. Among cardiac pathologies, AF is most often associated with mitral valve (MV) disease. Bipolar radio frequency (RF) AF ablation during MV surgery has been reported with excellent outcomes [4, 5]; however, data regarding the effectiveness of concomitant cryoablation are limited [6, 7]. The aim of this study was to assess the efficacy of concomitant cryoablation in patients scheduled for MV surgery.

## MATERIALS AND METHODS

### Ethical statement

This study was approved by the Institutional Review Board of E. Meshalkin National Medical Research Centre (approval number: 2020-32; approval date: 5 March 2020). Written informed consent of patients was waived as this was a retrospective analysis of anonymized data.

### Study design

All consecutive patients (aged ≥18 years) who underwent open-heart surgery and concomitant AF ablation in our centre between December 2012 and January 2020 were reviewed for this study. This retrospective study included patients with indications for MV surgery, in accordance with the European Society of Cardiology and the European Association for Cardio-Thoracic Surgery guidelines [8], and concomitant documented paroxysmal, persistent or longstanding persistent AF. The exclusion criteria were the use of radiofrequency energy for AF ablation and other cardiac surgery procedures, such as aortic valve and aortic surgery, myectomy for hypertrophic obstructive cardiomyopathy and coronary artery bypass grafting.

### Outcome measures

The primary endpoint was freedom from atrial arrhythmia recurrence, defined as a lack of any documented on a 24-h Holter monitor AF, atrial flutter (Afl) or atrial tachycardia episode lasting longer than 30 s. Secondary endpoints were the proportion of patients in sinus rhythm or in sinus rhythm off antiarrhythmic drugs, survival, presence of major adverse cardiovascular events (death, myocardial infarction, stroke, hospitalization for heart failure), rate of permanent pacemaker (PPM) implantation and need for catheter ablation due to atrial rhythm disturbance.

### Operative technique

**Figure 1:**
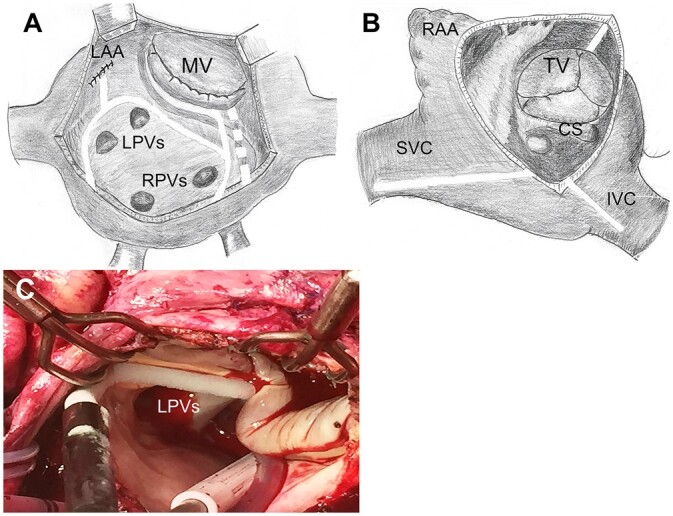
Cryoablation lesion set performed in the study population. (**A**) Left atrial lesions. (**B**) Right atrial lesions. (**C**) Intraoperative photography. The dotted line depicts epicardial line to the coronary sinus. CS: coronary sinus; IVC: inferior vena cava; LAA: left atrial appendage; LPVs: left pulmonary veins; MV: mitral valve; RAA: right atrial appendage; RPVs: right pulmonary veins; SVC: superior vena cava; TV: tricuspid valve.

Exposure of the MV in most procedures (70.7%) was performed using median sternotomy with standard cardiopulmonary bypass. In cases of minimally invasive MV (29.3%) surgery, cardiopulmonary bypass was established via femoral artery and vein cannulation, while the exposure was achieved with a 4- to 5-cm incision at the right anterolateral fourth intercostal space. Myocardial protection was provided using antegrade cold cardioplegia (Custodiol; Dr Kohler Pharma, Alsbach Hahnlein, Germany). The ablation procedure was performed using a CryoIce (AtriCure, Inc, Mason, OH, USA) N2O probe with an application time of 2 min for all lines. Left atrium (LA) pattern lesion set consisted of Box ‘U’ encircling lesion, line from left atrial appendage to ‘box’ and MV annulus line, which was supplemented with an additional epicardial line to the coronary sinus (Fig. 1). In cases of right atrium (RA) ablation, lines were applied to the tricuspid valve (TV) annulus, superior and inferior vena cava. The selection of lesion set was carried out based on surgeon preference. The LA appendage was excluded in all patients using an external 4/0 polypropylene suture in conventional cases or closed endocardial using two layers of sutures via a minimally invasive approach. LA appendage closure was confirmed using transoesophageal echocardiography in all patients.

### Postoperative management

After discharge, all patients were prescribed amiodarone 200 mg per day for 3 months. At 3 months after discharge, the first Holter monitoring was scheduled. In patients with sinus rhythm, class I/III antiarrhythmic medication was discontinued. For any case of documented AF, Afl or paroxysmal atrial tachycardia, amiodarone was prolonged to 6 months. In cases of non-restoration of sinus rhythm, the patient was consulted by an arrhythmologist about the need for catheter ablation.

Lifelong warfarin therapy was administered in cases of MV replacement with mechanical prostheses; in other cases, warfarin was prescribed for 6 months then discontinued in the absence of atrial arrhythmias episodes based on the results of 24-h Holter monitoring.

Patient heart rhythm control was assessed by 24-h Holter monitoring at 3, 6 and 12 months after surgery, then annually thereafter.

### Statistical analysis

Data analysis was performed using the STATA software for Windows, version 13.0 (StataCorp LP, College Station, TX, USA). Continuous data were presented as mean ± standard deviation or median (interquartile range) and categorical data were presented as absolute numbers (%). The Kaplan–Meier method was used to evaluate survival, freedom from atrial arrhythmias recurrence and thromboembolic events [presented with 95% confidence intervals (CI)]. Risk factor analysis of atrial arrhythmia recurrence was performed using the Cox proportional hazard regression method. The proportional hazard assumption was tested using Schoenfeld residuals. Logistic regression models were used to assess risk factors of thromboembolic events and PPM implantation. The analysis included factors such as age, sex, body surface area, New York Heart Association class, aetiology of MV pathology (rheumatic disease, degenerative disease, endocarditis), MV lesion (stenosis, insufficiency or mixed lesion), minimally invasive MV surgery, ablation set [biatrial (BA) or isolated LA], AF duration, TV surgery, surgeon experience (experience of ≥50 or <50 surgical ablation procedures), LA and RA sizes, left ventricular ejection fraction, pulmonary artery pressure, comorbidity (hypertension, thyroid disorders, diabetes mellitus, chronic obstructive pulmonary disease), bypass and aortic clamp time, and atrial arrhythmia paroxysm before discharge. Univariable analysis was initially performed. Variables with a value of *P* ≤ 0.2 in the univariate analyses and clinically relevant factors were included in the multivariable regression models. *P*-values of <0.05 in the multivariable analyses were used to determine significance.

## RESULTS

Two hundred and forty-two patients met study inclusion criteria (see Supplementary Material, Fig. S1). A total of 78 patients (32.2%) presented with paroxysmal, 50 (20.7%) with persistent AF and 114 (47.1%) had longstanding persistent AF. Other preoperative patient characteristics are summarized in Table 1.

**Table 1:** Baseline patient characteristics

	*n* = 242
Age (years)	54.8 ± 0.65
Male, *n* (%)	104 (43.0)
BMI (kg/$m^{2})$	27.2 ± 0.32
NYHA class, *n* (%)	
NYHA I	5 (2.1)
NYHA II	70 (28.9)
NYHA III	167 (69.0)
NYHA IV	0
AF type, *n* (%)	
Paroxysmal AF	78 (32.2)
Persistent AF	50 (20.7)
Longstanding AF	114 (47.1)
AF duration (months)	43.1 ± 3.72
Permanent pacemaker, *n* (%)	4 (1.6)
Aetiology, *n* (%)	
Rheumatic heart disease	148 (61.2)
Degenerative disease	90 (37.2)
Endocarditis	4 (1.6)
Mitral valve lesion, *n* (%)	
Stenosis	58 (24.0)
Insufficiency	108 (44.6)
Mixed lesions	76 (31.4)
Tricuspid valve pathology, *n* (%)	124 (51.2)
Insufficiency ≥ moderate grade	120 (49.6)
Mixed lesions or stenosis	4 (1.6)
Echocardiography data	
Left atrium (cm)	6.6 ± 0.05
Right atrium (cm)	5.8 ± 0.05
Left ventricle ejection fraction (%)	61.0 ± 0.62
Systolic pulmonary artery pressure (mmHg)	46,3 ± 0.75
Comorbidity, *n* (%)	
Hypertension	107 (44.2)
Thyroid disorders	42 (17.4)
Diabetes mellitus	11 (4.5)
Chronic obstructive pulmonary disease	6 (2.5)

Data are presented as means ± standard deviations, numbers (%).

AF: atrial fibrillation; BMI: body mass index; NYHA: New York Heart Association.

### Intraoperative results

MV repair procedures were performed in 93 (38.4%) cases. Isolated LA lesion set was accomplished in 105 (43.4%) patients and BA lesion set in 137 (56.6%) patients. More than half of the procedures were supplemented by TV intervention (124; 51.2%; Table 2).

**Table 2:** Operative data

Operative data	n = 242
Bypass time (min)	137.7±3.9
Aortic clamping time (min)	96.0±2.3
MV replacement, *n* (%)	149 (61.6)
Mechanical valve prosthesis	140 (94.0)
Biological valve prosthesis	9 (6.0)
MV repair, *n* (%)	93 (38.4)
Minimally invasive MV surgery, *n* (%)	71 (29.3)
Ablation set, *n* (%)	
BA ablation	137 (56.6)
LA ablation	105 (43.4)
TV surgery, *n* (%)	124 (51.2)
Replacement	8 (6.5)
Repair	116 (93.5)

Data are presented as means ± standard deviations, numbers (%).

BA: biatrial; LA: left atrial; MV: mitral valve; TV: tricuspid valve.

### Early morbidity and mortality

In the early postoperative period, one (0.4%) patient died due to acute heart failure. Re-exploration for bleeding was required in 15 (6.2%) cases. In terms of other complications, five (2.1%) patients had acute coronary syndrome, three (1.2%) had cerebral ischaemia events (two strokes and one transient ischaemic attack) and three (1.2%) suffered from superficial wound infection.

### Survival

The mean follow-up period duration was 43.9 ± 23.8 months. Follow-up data were available for 216 (89.6%) patients.

**Figure 2:**
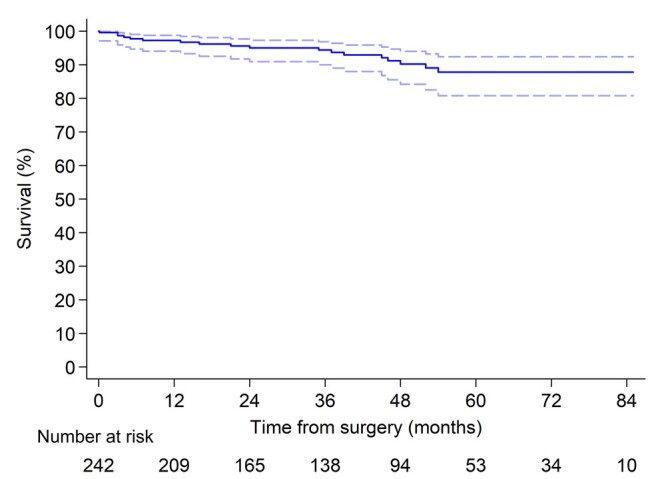
Survival of patients underwent concomitant cryoablation during mitral valve surgery.

There were 17 late deaths, 7 cases were due to stroke, in 5 patients, the reason for death was myocardial infarction, 3 died due to progressive heart failure and 2 deaths were non-cardiac in origin (oncology and complication from the flu). The survival rates at 1, 3 and 5 years were 97.3% (95% CI, 93.9–98.8), 94.3% (95% CI, 89.9–96.9) and 87.7% (95% CI, 80.5–92.3%), respectively (Fig. 2).

### Rhythm outcomes

In-hospital AF paroxysm was observed in 50 (20.7%) patients; 19 (38%) patients underwent electrical cardioversion. A total of 206 (out of 241, 85.5%) patients were discharged with sinus rhythm, 19 (7.9%) with AF/Afl and 16 (6.6%) with PPM (in 4 of which the PPM was implanted previously).

**Figure 3:**
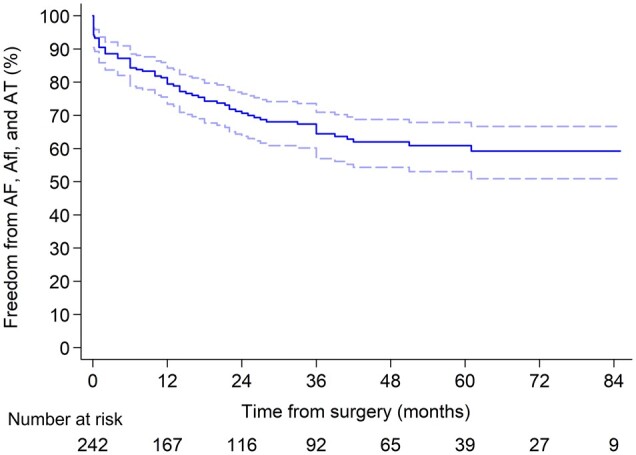
Freedom from atrial arrhythmia paroxysms. AF: atrial fibrillation; Afl: atrial flutter; AT: atrial tachycardia.

**Figure 4:**
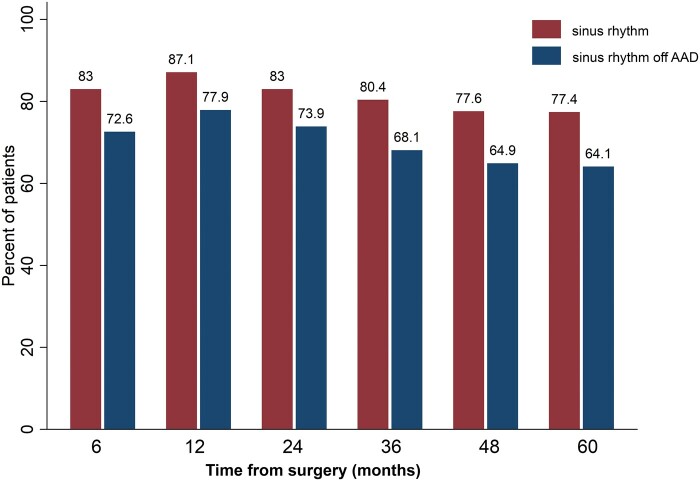
Per cent of patients in sinus rhythm and sinus rhythm off antiarrhythmic drugs. AAD: antiarrhythmic drug.

During the follow-up period, atrial arrhythmia developed in 59 more patients. The most common occurrence was AF in 35 patients, then Afl in 22 and paroxysms of atrial tachycardia in 2 cases. Freedom from atrial arrhythmia paroxysms at 1, 3 and 5 years were 79.0% (95% CI, 72.9–83.9), 64.0% (95% CI, 56.5–70.6) and 60.5% (95% CI, 52.6–67.5), respectively (Fig. 3). Return to sinus rhythm regardless of antiarrhythmic drugs at 1, 3 and 5 years was 87.1%, 80.4% and 77.4%, respectively. Sinus rhythm off antiarrhythmic drugs at 1, 3 and 5 years was 77.9%, 68.1% and 64.1%, respectively (Fig. 4).

At follow-up, 70.4% (152 out of 216) of patients continued to receive oral anticoagulation due to mechanical prosthesis and/or arrhythmia recurrence. Only 54% of the international normalized ratio values made during follow-up were within the therapeutic target value. Self-management of oral anticoagulation was used only by 17 (11.2%) patients.

In the univariate Cox hazard regression model, persistent AF (*P* = 0.011), larger baseline RA size (*P* < 0.001), lower surgeon experience (*P* < 0.001), TV surgery (*P* = 0.001), hypertension (*P* = 0.018), hospital AF paroxysms (*P* < 0.001) and MV surgery using the standard approach (*P* < 0.001) were associated with atrial arrhythmia recurrence (Table 3). However, multivariable Cox regression analysis showed that isolated LA lesion set [hazard ratio (HR) = 1.83, *P* = 0.038], RA size (HR = 1.63, *P* = 0.002), surgeon experience (HR = 0.31, *P* = 0.003) and AF paroxysms in early postoperative period (HR = 2.09, *P* = 0.002) remained significant risk factors for recurrent atrial arrhythmia.

**Table 3:** Risk factors for atrial arrhythmia recurrent (Cox hazard regression model)

Risk factor	Univariable model		Multivariable model	
	HR (95% CI)	*Р*-value	HR (95% CI)	*Р*-value
Sex (men)	0.76 (0.48–1.23)	0.27		
Rheumatic lesion	1.42 (0.87–2.31)	0.16	1.00 (0.52–1.95)	0.99
Persistent AF	2.01 (1.17–3.46)	0.011	1.23 (0.68–2.21)	0.49
AF duration (months)	1.00 (0.99–1.00)	0.47		
LA size (mm)	1.14 (0.88–1.48)	0.32		
RA size (mm)	1.81 (1.37–2.40)	<0.001	1.63 (1.19–2.23)	**0.002**
LVEF (%)	0.98 (0.96–1.0)	0.11	0.99 (0.97–1.02)	0.51
Minimally invasive MV surgery	0.26 (0.14–0.49)	<0.001	0.58 (0.27–1.24)	0.16
Surgeon experience	0.25 (0.12–0.53)	<0.001	0.31 (0.15–0.66)	**0.003**
LA lesion set	1.63 (0.93–2.86)	0.087	1.83 (1.03–3.25)	**0.038**
TV surgery	2.32 (1.43–3.78)	0.001	1.43 (0.82–2.49)	0.21
Hypertension	1.74 (1.10–2.75)	0.018	1.15 (0.69–1.91)	0.59
Thyroid disorders	1.63 (0.96–2.78)	0.072	1.29 (0.74–2.28)	0.37
MV replacement	1.36 (0.84–2.19)	0.21	0.83 (0.48–1.43)	0.49
Hospital AF paroxysms	2.66 (1.68–4.23)	<0.001	2.09 (1.30–3.35)	**0.002**

AF: atrial fibrillation; CI: confidence interval; HR: hazard ratio; LA: left atrium; LVEF: left ventricular ejection fraction; MV: mitral valve; RA: right atrium; TV: tricuspid valve.

Bold values represent statistically significant *P*-values (*P*<0.05).

In addition, risk factors for AF and Afl paroxysm were separately assessed. Multivariable Cox regression analysis showed that larger RA diameter (HR = 2.53, *P* = 0.001) was a significant predictor only for Afl recurrence, not for AF (HR = 1.33, *P* = 0.16).

There were 30 cases of hospitalization for rhythm-related interventions during the follow-up period. Catheter ablation to maintain sinus rhythm was performed in 11 (4.6%) patients (one case of paroxysmal atrial tachycardia and 10 cases of Afl). In Afl cases, six patients presented with typical Afl and four presented with atypical post-incisional re-entry.

### Pacemaker implantation

In the early postoperative period, 12 (4.9%) patients required PPM implantation: 5 (41.7%) due to atrioventricular block and 7 (58.3%) due to sinus node dysfunction. The rates of PPM implantation for BA and LA lesion sets were 5.8% and 3.8%, respectively (*P* = 0.47). During the follow-up, four more patients required PPM (three due to sinus node dysfunction and one due to Frederick syndrome). Multivariable logistic regression model revealed TV surgery (odds ratio = 3.52, *P* = 0.041) was a significant risk factor for PPM implantation (Supplementary Material, Table S1).

### Stroke and thromboembolic events

**Figure 5:**
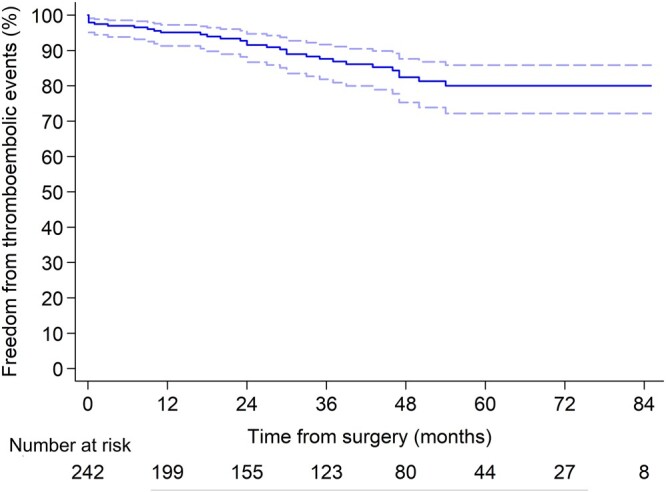
Freedom from thromboembolic events.

Thromboembolic events occurred in 27 patients during the long-term follow-up period: 13 had strokes, 11 had transient ischaemic attacks and 3 had systemic embolism. Freedom from thromboembolic events at 1, 3 and 5 years was 95.0% (95% CI, 91.1–97.2), 87.5% (95% CI, 81.6–91.6) and 79.7% (95% CI, 71.7–85.6%), respectively (Fig. 5). Thromboembolic events rate was 3.7% per patient-year.

Moreover, 5 patients had haemorrhagic strokes. Freedom from all strokes and transient ischaemic attacks at 1, 3 and 5 years was 95.0% (95% CI, 90.7–97.1), 86.9% (95% CI, 81.0–91.0) and 78.3% (95% CI, 70.2–84.5%), respectively.

In the multivariable logistic regression model, longer aortic clamping time (odds ratio = 1.01, *P* = 0.005) and MV replacement (odds ratio = 2.45, *P* = 0.037) were significant risk factors for strokes and thromboembolic events (Supplementary Material, Table S2).

Recurrent significant mitral regurgitation after MV repair occurred in three (1.2%) patients due to ring dehiscence in two patients and calcification of the posterior valve leaflet in one patient. All patients underwent successful MV replacement.

## DISCUSSION

This study demonstrates that concomitant cryoablation during MV surgery is associated with an acceptable safety profile and freedom from atrial arrhythmias recurrences for up to five years. Moreover, the BA lesion set is a factor associated with better sinus rhythm retention at follow-up and surgeon’s experience significantly influenced AF ablation success.

According to current guidelines, concomitant surgical ablation for AF is recommended at the time of MV procedures as it does not increase operative risk and, at the same time, sinus rhythm restoration demonstrates survival benefit, improved symptoms and long-term quality of life [2, 3]. The original ‘cut and sew’ technique, proposed in 1987, has shown high efficiency; however, it did not find widespread adoption due to technical difficulty since it required prolonged cardiopulmonary bypass times, as well as an increased possibility of complete atrioventricular block among other complications. Currently, ablation with the use of alternative energy sources has supplanted the ‘cut and sew’ technique. Among these alternative energy sources, RF and cryoablation are the most used. In a systematic review by Khargi *et al.* [9], no significant difference in postoperative sinus rhythm conversion rates between ‘cut and sew’ and alternative ablation groups was found; RF and cryothermal energy sources were characterized by greater simplicity and convenience for the surgeon, less invasiveness and lower complication incidence.

Results of concomitant AF ablation during cardiac surgery vary significantly across studies, due to differences in patient populations, applied lesion lines, varied combination of energy sources and different approaches to follow-up rhythm assessment. Freedom from atrial arrhythmia recurrence after RF and cryoablation during MV surgery ranges from 61% to 98% at 1 year and from 57% to 89% at 5 years [4–6, 10–18]. In this study, the rate of hospital discharge on AF/Afl was 7.9%, which is comparable with that in other studies [12]. According to Niv Ad’s data, hospital AF recurrence occurred in 52% of the no-amiodarone group and 19% of the group received amiodarone after surgical ablation [19]. In our study, the rate of AF paroxysms during blanking period is quite acceptable (20.7%) and corresponds to above-mentioned results. We found that 1- and 5-year rates of freedom from atrial arrhythmia paroxysm after AF ablation using cryoprobe alone were 78.5% and 60%, respectively, which is slightly lower than that in some similar studies. The reason for this difference can be explained by patient baseline characteristics. In our study, the patients had longer AF duration, larger LA and more frequent rates of rheumatic MV disease [5]. Moreover, in some papers [6] electrocardiogram, instead of Holter monitoring, was used for rhythm assessment; therefore, only persistent AF forms were identified, while paroxysmal forms might not have been documented.

There are limited and controversial literature data comparing cryothermal and RF energy-based ablation techniques. There are no direct prospective randomized studies comparing RF and cryoablation; therefore, there is a lack of reliable information on the best energy source or lesion set. Several retrospective studies comparing RF and cryothermal energy sources in MV patients found no significant difference in their safety and effectiveness in maintaining sinus rhythm [13, 14]. Ad *et al.* [15] showed advantages of cryothermal energy alone over a combination of cryothermal and bipolar RF ablation in sinus rhythm restoration and stroke reduction, which the authors explained as a possible limitation of the bipolar RF algorithms, developed on the basis of tests on healthy atrial tissue in animals.

Large LA size, longer history of AF and isolated LA ablation are the most commonly reported predictors of atrial arrhythmia recurrence after AF ablation [5, 7, 12, 14, 15]. Other predictors reported include patient age, reduced left ventricular ejection fraction [7, 14], lower surgeon experience [5], presence of early postoperative AF events, higher pulmonary artery pressure [12], residual/recurrent MV disease in the postoperative period, presence of F-wave on electrocardiogram, hypertension and chronic obstructive pulmonary disease.

The most effective surgical ablation lesion set remains a decision-making challenge for surgeons. Previously, a small number of randomized trials showed no difference between BA and LA ablation in terms of arrhythmia recurrence [16]. More recently, the BA lesion set demonstrated benefits over isolated LA for long-term sinus rhythm maintenance [17, 18, 20–23]. Recurrent Afl is observed in 13–21% of patients undergoing LA ablation [24]. However, there are data that additional right atrial lesions are associated with an increased risk of sinus node dysfunction and PPM implantation [21]. Recent guidelines state that MV patients with advanced disease (long AF duration and enlarged LA size) should undergo a BA surgical ablation [2, 3]. In this study, the BA lesion also was associated with rhythm success at long-term follow-up. These findings agree with data obtained in our previous propensity score matching study on BA versus LA ablation for persistent AF treatment [18].

Enlargement of the RA was a risk factor for Afl paroxysms at follow-up in our study. The rate of Afl among the observed atrial arrhythmias was high (37.3%), and mapping during catheter ablation revealed that typical Afl occurs more often. This confirms the need to perform right atrial ablation in the case of right chamber dilatation and TV diseases. In contrast, LA dilatation was not associated with arrhythmia recurrence, possibly because a vast number of patients had significant atrial dilatation (>60 mm) and the standard deviation of the mean was small.

Concomitant AF ablation during MV surgery increases the risk of PPM implantation in comparison with an MV procedure alone, although there is no evidence that this adversely affects long-term survival [22]. The incidence of PPM implantation after AF ablation during cardiac surgery varies significantly between 5% and 25%, with the most reported risk factors for pacemaker implantation being the BA lesion set and longer AF duration [16, 22, 23]. In a meta-analysis by Phan *et al.* [17], PPM implantation rate was significantly higher in the BA versus LA group (7.0% and 5.4%, respectively). In a recent meta-analysis including 28 studies and 7065 patients [23], performing a BA approach was associated with a 1.8‐fold increase in PPM implantation (7.1% vs 5.4%, *P* < 0.0001) and a three‐fold increase of sinoatrial node dysfunction (4.9% vs 1.7%, *P* = 0.002) compared with LA ablation. According to literature data, there is no difference between cryothermal and RF energy sources in terms of PPM implantation rate [22]. In our previous study [18] that included a small proportion of cryoablation patients, BA lesion set and longer AF duration were associated with PPM need, with a 16.5% and 7.5% rate in the BA and LA groups, respectively. However, in this study, the incidence of PPM implantation did not differ between BA and LA approaches (5.8% and 3.8%, respectively, *P* = 0.47); the lesion set was not a PPM predictor. The lower PPM rate in this study can be explained by surgical experience accumulation and some changes in the RA lesion set, committed after years of previous RF ablation practice. The lines connected with right atrial appendage (from the appendage to the tricuspid annulus and lateral wall line) were excluded from lesion set pattern. Furthermore, we started doing superior vena cava line 6–8 mm lower than we did earlier.

An advantage of cryoablation is its convenience during minimally invasive cardiac surgery. Previous studies have shown comparable results of bipolar RF ablation through full sternotomy and right mini-thoracotomy [25]. The difference between our study and previous reports is that, in all cases of conventional or minimally invasive approaches, we performed standardized lesion lines using a cryoprobe alone. Our study shows that concomitant cryoablation can be efficiently performed via minimally invasive right thoracotomy with results comparable to cryoablation via standard sternotomy.

In this study, the rates of embolic and haemorrhagic cerebral complications were high. According to literature, MV replacement in comparison with repair is a risk factor for thromboembolic complications [26, 27], which was confirmed in this study. The reason for a high rate of strokes in our study is the high proportion of patients who received a mechanical prosthesis and the unsatisfactory quality of anticoagulation control during follow-up. Anticoagulation self-management significantly decreases the rate of these complications and improves long-term outcomes [28].

### Limitations

The first limitation of this study is its retrospective design. Second, the study presents the results of a single-centre analysis; procedures were performed by several surgeons, which can lead to differences in results. Nevertheless, this reflects real clinical practice. Third, some patients were lost at follow-up (∼10%). Fourth, we used 24-h Holter monitoring instead of continuous loop recording or 72-h Holter monitoring. Fifth, we monitored the closure of the left atrial appendage using only intraoperative transoesophageal echocardiography and did not control late residual exclusion and thrombus formation in the postoperative period. Sixth, the mean follow-up duration was relatively short. Finally, cryoablation has not been compared with other energy sources, such as bipolar RF ablation. Not all patients with recurrence of atrial arrhythmia had an electrophysiological study, resulting in a lack of defined mechanisms for failure in some cases.

## CONCLUSION

Concomitant cryoablation during MV surgery is a safe and reproducible technique. The procedure provides acceptable freedom from atrial arrhythmias recurrences during long-term follow-up. The BA lesion set has advantages over the LA pattern in terms of atrial arrhythmias freedom. Surgeon experience significantly influences AF ablation success. MV replacement by mechanical valve prosthesis is associated with a high risk of thromboembolic complications. Randomized clinical trials are needed to compare RF and cryoablation.

## SUPPLEMENTARY MATERIAL

Supplementary material is available at *ICVTS* online.

**Conflict of interest:** none declared. 

### Data Availability Statement

Data available on request.

### Author contributions

**Alexander Bogachev-Prokophiev:** Conceptualization; Methodology; Supervision. **Ravil Sharifulin:** Software; Writing—review & editing. **Anastasiia Karadzha:** Data curation; Writing—original draft. **Sergey Zheleznev:** Writing—review & editing. **Alexander Afanasyev:** Investigation; Writing—review & editing. **Mikhail Ovcharov:** Data curation. **Alexey Pivkin:** Resources; Software; Visualization. **Anton Zalesov:** Data curation. **Sergey Budagaev:** Data curation. **Sergey Ivantsov:** Visualization. **Alexander Chernyavsky:** Conceptualization; Supervision; Validation.

### Reviewer information

Interactive CardioVascular and Thoracic Surgery thanks Ko Bando, Stefano Benussi, Leonardo Paim and the other anonymous reviewers for their contribution to the peer review process of this article.

## Supplementary Material

ivab322_Supplementary_Data

## Notes

Presented at the 34th Annual Meeting of the European Association for Cardio-thoracic Surgery, Barcelona, Spain, 8–10 October 2020.

## ABBREVIATIONS

AF: Atrial fibrillation
Afl: Atrial flutter
BA: Biatrial
CI: Confidence interval
HR: Hazard ratio
LA: Left atrium
MV: Mitral valve
PPM: Permanent pacemaker
RA: Right atrium
RF: Radio frequency
TV: Tricuspid valve
