# Supplementary material for: Results of concomitant cryoablation for atrial fibrillation during mitral valve surgery
Source: Interact Cardiovasc Thorac Surg. 2021 Nov 13;34(4):540–7. doi: 10.1093/icvts/ivab322 (PMC8972332; doi:10.1093/icvts/ivab322)
Supplement: ivab322_Supplementary_Data [file ivab322_supplementary_data.zip › Supplement tables.docx]

**Supplementary Table S1:** Risk factors for permanent pacemaker implantation (logistic regression model)

| Risk factor | Univariable model | | Multivariable model | |
| --- | --- | --- | --- | --- |
|  | OR (95% CI) | Р value | OR (95% CI) | Р value |
| Persistent AF | 3.83 (0.85-17.16) | 0.080 | 3.34 (0.72-15.48) | 0.12 |
| TV surgery | 3.22 (1.02-10.18) | 0.046 | 3.52 (1.05-11.74) | 0.041 |
| Surgeon | 1.27 (0.43-3.77) | 0.66 | - | - |
| AF duration | 0.99 (0.98-1.00) | 0.43 | - | - |
| LA lesion set | 0.65 (0.24-1.76) | 0.39 | - | - |
| MV replacement | 1.43 (0.49-4.20) | 0.52 | - | - |

AF, atrial fibrillation; LA, left atrial; MV, mitral valve; PAP, pulmonary artery pressure; TV, tricuspid valve; OR: odds ratio; CI: confidence interval.

**Supplementary Table S2:** Risk factors for strokes and thromboembolic events (logistic regression model)

| Risk factor | Univariable model | | Multivariable model | |
| --- | --- | --- | --- | --- |
|  | OR (95% CI) | Р value | OR (95% CI) | Р value |
| Sex (men) | 1.59 (0.78-3.24) | 0.20 | 2.01 (0.91-4.46) | 0.084 |
| Age | 1.01 (0.97-1.04) | 0.67 | - | - |
| History of stroke | 1.23 (0.39-3.85) | 0.72 | - | - |
| Atrial arrhythmia recurrent | 1.34 (0.64-2.82) | 0.44 | 1.94 (0.86-4.38) | 0.11 |
| Permanent pacemaker | 2.61 (0.86-7.91) | 0.091 | 2.76 (0.89-8.57) | 0.080 |
| LA lesion set | 1.10 (0.45-2.71) | 0.83 | - | - |
| LVEF | 0.97 (0.94-1.01) | 0.16 | 0.98 (0.94-1.02) | 0.25 |
| Aortic clamping time | 1.01 (1.00-1.02) | 0.015 | 1.01 (1.00-1.02) | 0.005 |
| MV replacement | 1.90 (0.85-4.26) | 0.12 | 2.45 (1.05-5.69) | 0.037 |
| Minimal invasive MV surgery | 1.91 (0.92-3.97) | 0.082 | 1.68 (0.63-4.50) | 0.30 |

LVEF, left ventricular ejection fraction; MIMV, minimally invasive mitral valve; PPM, permanent pacemaker; MV, mitral valve; OR: odds ratio; CI: confidence interval.
